# Zirconium‐Rich Strategy in Ultrathin Hf_0.5_Zr_0.5_O_2_ toward Back‐End‐of‐Line‐Compatible Ferroelectric Random Access Memory

**DOI:** 10.1002/advs.202509384

**Published:** 2025-08-14

**Authors:** Yinchi Liu, Jiajia Tao, Xiaoyu Dou, Kangli Xu, Yuchun Li, Handong Zhu, Hongliang Lu, Yanxi Li, Chi Liu, Jiezhi Chen, Lin Chen, Shijin Ding, Jixuan Wu, Wenjun Liu

**Affiliations:** ^1^ College of Integrated Circuits and Micro‐Nano Electronics Fudan University Shanghai 200433 China; ^2^ School of Microelectronics Fudan University Shanghai 200433 China; ^3^ Zhangjiang Laboratory Shanghai 201210 China; ^4^ School of Information Science and Engineering Shandong University Jinan 250100 China; ^5^ Shenyang National Laboratory for Materials Science Institute of Metal Research Chinese Academy of Sciences Shenyang 110000 China; ^6^ Shaoxin Laboratory Shaoxing 312000 China

**Keywords:** back‐end‐of‐line, ferroelectricity, reliability, tensile strain, zirconium‐rich layer

## Abstract

HfO_2_‐based ferroelectric devices have garnered lots of attention in embedded memory due to its exceptional complementary metal oxide semiconductor (CMOS) compatibility as well as sub‐10 nm scalability. Nevertheless, challenges such as double remanent polarization (2*P*_r_) degradation and thermal budget issues while scaling the Hf_0.5_Zr_0.5_O_2_ (HZO) thickness have limited its integration in high‐intensity memory and high‐speed computing. Here, an effective strategy involving the zirconium‐rich layer (Zr‐RL) is developed to address this dilemma. Remarkably low operating voltage of 1.0 V, alongside exceptional ferroelectricity with 2*P*_r_ of 43.4 *µ*C cm^−2^ and a coercive voltage of 0.75 V are achieved in the ferroelectric capacitor with sub‐6 nm HZO/Zr‐RL/HZO stack. First‐principles calculations reveal that the incorporation of Zr‐RL induces a 0.76% tensile strain, which effectively reduces the growth barrier and surface energy of the ferroelectric phase, thereby facilitating the crystallization of the HZO/Zr‐RL/HZO stack under a low thermal budget. Moreover, robust reliability, including a high breakdown voltage of 3.69 V, superior endurance characteristics exceeding 10^11^ cycles, and excellent retention time of 10 years are demonstrated in the ferroelectric capacitor with HZO/Zr‐RL/HZO stack. Our investigations provide new insights into building a low‐voltage operation, high ferroelectricity and reliability, long data retention, and back‐end‐of‐line‐compatible ferroelectric random access memory in scaled CMOS technology nodes.

## Graphical abstract

Here, the zirconium‐rich layer (Zr‐RL) strategy is developed in terms of ferroelectric (FE) properties, microstructure, switching kinetics, and reliability. Compared to the FE capacitor with conventional HZO film, the sub‐6 nm HZO/Zr‐RL/HZO stack exhibits a significantly low annealing temperature, an ultra‐low Vop, and an excellent ferroelectricity. First‐principles calculations reveal at the atomistic level that the Zr‐RL could introduce the additional tensile strain and reduce the growth barrier of FE phase.

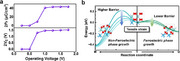

## 1 Introduction

The growing demands for high‐speed computing and high‐density memory are rendering the conventional Von Neumann architecture increasingly inadequate to meet the requirements of next‐generation technologies. To circumvent these issues, considerable attention has been directed toward the advancement of neuromorphic devices as well as in‐memory computing technologies, which enable more efficient data processing.^[^ ^1^, ^2^ ^]^ The memory devices based on Zr‐doped HfO_2_ (Hf_0.5_Zr_0.5_O_2_, HZO) ferroelectric (FE) materials, including ferroelectric tunnel junctions (FTJ), ferroelectric random access memories (FeRAM), and ferroelectric field‐effect transistors (FeFET), have garnered widespread attention in embedded memory and monolithic 3D integrated in‐memory computing chips owing to their high complementary metal oxide semiconductor (CMOS) compatibility, low power consumption, fast switching speed, and superior scalability.^[^ ^3^, ^4^ ^]^ However, the typical thickness of ≈10 nm and the high coercive voltage (*V*_c_) of nearly 1.2 V result in excessive operating voltages (*V*_op_) for HZO‐based FE memories, necessitating a further reduction to below 1.2 V for meeting the power driving requirements in advanced process nodes.^[^ ^5^, ^6^, ^7^ ^]^ Additionally, the excessive high *V*_op_ leads to the premature hard breakdown during the erase/write cycles, limiting the reliability improvement.^[^ ^8^, ^9^, ^10^ ^]^ A direct approach to reduce *V*_op_ is to shrink the thickness of FE films. However, this reduction in thickness increases the phase formation barriers, necessitating a rise in the annealing temperature (> 450 °C) for maintaining a considerable remanent polarization (*P*_r_), which is incompatible with the back‐end of line (BEOL) process.^[^ ^11^ ^]^ It is widely accepted that the origin of ferroelectricity in HZO films is rooted in the metastable orthorhombic (O‐) phase with the *Pca*2_1_ space group. The formation of this stable O‐phase primarily results from the strain predominantly influenced by the processing parameters, such as the lattice mismatch of HZO to the electrodes, coefficient of thermal expansion difference between HZO and substrate, the capping effect from the top electrode, the densification of the HZO film during cooling, etc.^[^ ^12^, ^13^, ^14^, ^15^, ^16^ ^]^ Previous studies have demonstrated the impact of local stress on the 2*P*_r_, *V*_c_, and reliability of FE capacitors through approaches such as electrode engineering,^[^ ^17^ ^]^ superlattice structures,^[^ ^18^ ^]^ interface layer engineering,^[^ ^3^ ^]^ and elemental doping.^[^ ^19^ ^]^ For example, Cui et al. revealed that varying the number of ZrO_2_ and HfO_2_ cycles in superlattice structures introduces different local thermal stress mismatches. This additional stress results in an earlier saturation of polarization during the annealing process and thus less annealing time.^[^ ^18^ ^]^ Kashir et al. presented the reduced *V*_c_ and dielectric permittivity in Hf_0.5_Zr_0.5_O_2_/ZrO_2_ thin films, verifying the higher mobility of domain wall in Hf_0.5_Zr_0.5_O_2_/ZrO_2_ thin films.^[^ ^20^ ^]^ Jang et al. developed a Hf_1‐x_Zr_x_O_2_ ferroelectric capacitor with a nanolaminate structure that operated at remarkably low voltages, showing excellent retention and endurance.^[^ ^21^ ^]^ Therefore, introducing additional tensile strain is a promising strategy to enhance the ferroelectricity and reliability of ultrathin FE films under a low thermal budget, as it reduces the barriers for FE‐phase formation and increases the O‐phase components. Nevertheless, systematic studies addressing these issues have yet to be reported.

Herein, we propose the zirconium‐rich layer (Zr‐RL) strategy to achieve a saturated 2*P*_r_ of 43.4 *µ*C cm^−^^2^ at an ultra‐low *V*_op_ of 1.0 V in the ultrathin FE film which is compatible with the BEOL process. The mechanism for optimizing the ferroelectricity and reducing thermal budget is analyzed through first‐principles calculations, which reveal that the introduction of Zr‐RL induces the tensile strain, thereby lowering the growth barriers of the FE phase. Additionally, the switching kinetics are simulated by nucleation‐limited switching (NLS) model and the cycling characteristics under the saturated frequency are measured. Excellent endurance is achieved, with over 10^10^ cycles at room‐temperature and over 10^7^ at 125 °C under 1.0 V at a frequency of 100 kHz, while the capacitor with HZO/Zr‐RL/HZO stack also exhibits a retention time of over 10 years under 1.0 V at 125 °C.

## 2 Result and Discussions

### 2.1 Fabrication and Ferroelectric Characteristics

**Figure 1:**
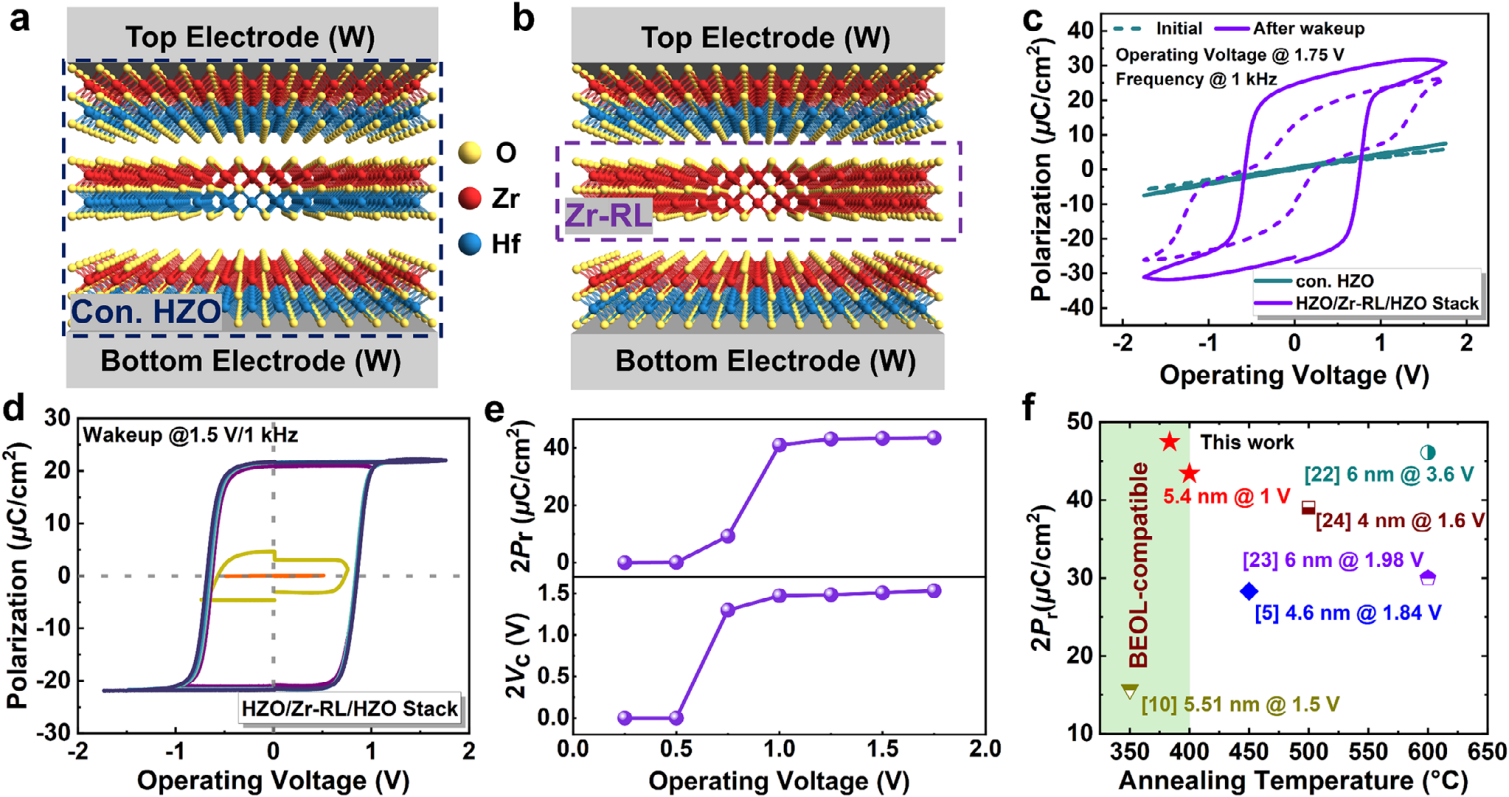
Ferroelectric switching with the low *V*_op_ and low thermal budget in ultrathin ferroelectric film using the Zr‐RL strategy. Schematic structures of the FE capacitors with a) conventional HZO and b) HZO/Zr‐RL/HZO stack. c) The *P–V* curves of initial and after 10^3^ wakeup cycles for the FE capacitors with conventional HZO and HZO/Zr‐RL/HZO stack annealed at 400 °C under the operating voltage of 1.75 V. d) The *P–V* loops after wakeup of FE capacitor with HZO/Zr‐RL/HZO stack annealed at 400 °C measured by PUND method. The wakeup process is implemented using square‐wave with an amplitude of 1.5 V, a frequency of 1 kHz, and a total of 10^3^ cycles. e) The extracted 2*P*_r_ and 2*V*_c_ as a function of *V*_op_ from the PUND measurement for the FE capacitor with HZO/Zr‐RL/HZO stack annealed at 400 °C. f) Benchmark plot comparing the reported approaches with our strategy for the FE capacitors with ultrathin HfO_2_‐based ferroelectric thin film.

The FE capacitors with the conventional HZO and HZO/Zr‐RL/HZO stack were fabricated using the plasma enhanced atomic layer deposition (PEALD) at a substrate temperature of 250 °C (see “Experimental Section” and Figure S1a,b (Supporting Information). **Figure** **1**a,b show the schematic structures of FE capacitors with the conventional HZO and HZO/Zr‐RL/HZO stack film, respectively. Figure 1c presents the polarization–voltage (*P–V*) curves under the *V*_op_ of 1.75 V of the FE capacitors with conventional HZO and HZO/Zr‐RL/HZO stack before and after a wakeup process. Unlike the FE capacitor with conventional HZO film, a competitive ferroelectricity is obtained in the FE capacitor with HZO/Zr‐RL/HZO stack. The wakeup process is implemented using square‐wave with an amplitude of 1.5 V, a frequency of 1 kHz, and a total of 10^3^ cycles. The detailed mechanism will be discussed later. To facilitate a more accurate observation of the FE polarization at different voltages, the positive‐up‐negative‐down (PUND) measurement was conducted as shown in Figure 1d. Rapid polarization switching occurred at an ultra‐low voltage of 1.0 V, and an excellent saturated 2*P*_r_ of ≈43.4 *µ*C cm^−2^ with the 2*V*_c_ of ≈1.5 V is achieved under the *V*_op_ of 1.75 V (Figure 1e). Utilizing the annealing temperature and 2*P*_r_ as performance indicators, Figure 1f provides a comparative analysis of ferroelectricity of FE capacitors with ultrathin FE film as reported in literature.^[^ ^5^, ^10^, ^22^, ^23^, ^24^ ^]^

### 2.2 Atomistic Insight into the Phase Structure

**Figure 2:**
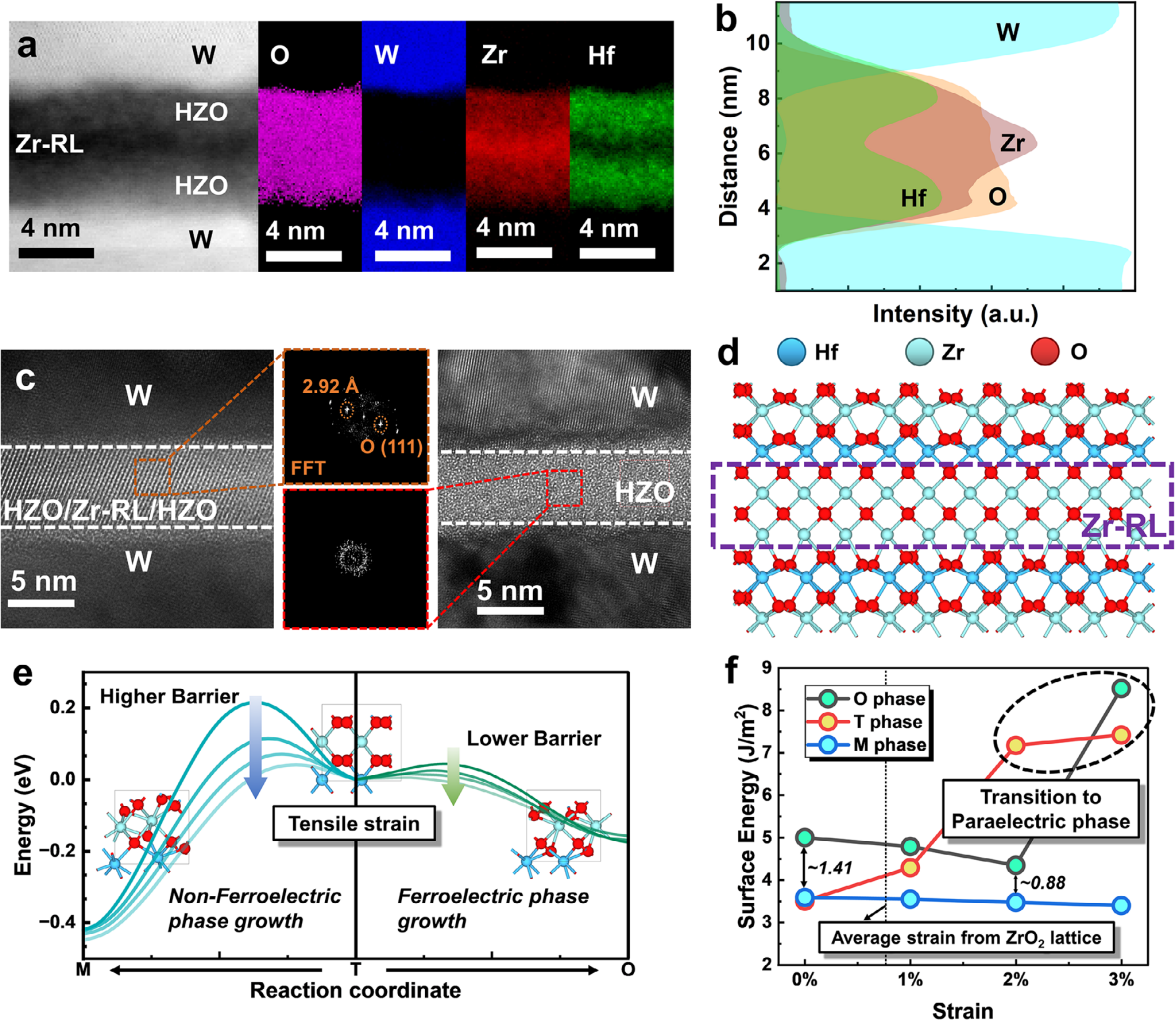
Intrinsic mechanism of low‐temperature crystallization via first‐principles calculations. a) High‐angle annular dark‐field (HAADF) STEM image and corresponding EELS elemental mapping of O, W, Zr, and Hf for the FE capacitor with HZO/Zr‐RL/HZO stack. b) Line profiles of EELS intensities for O, W, Zr, and Hf as a function of distance. c) Cross‐sectional TEM images of FE capacitors with conventional HZO and HZO/Zr‐RL/HZO stack annealed at 400 °C. The insets show the fast Fourier transform images of the O‐phase. d) The simulated structure of the HZO/Zr‐RL/HZO stack used in first‐principles calculations. e) Energy landscape for phase transition calculated by linear interpolation along the pathway of T→O and T→M. The transition barrier for T→O is much lower than that for T→M. f) The surface energy evolution of O‐, T‐, and M‐ phase as a function of strain. The average strain from ZrO_2_ lattice is 0.76%.

**Figure** **2**a shows the results of electron energy loss spectroscopy (EELS) analysis for the capacitor with the HZO/Zr‐RL/HZO stack. As shown in Figure 2a, the elemental mapping of O, W, Zr, and Hf reveals a clear elemental separation across the stack. The Zr‐RL is distinctly resolved between the two HZO layers, while the tungsten (W) electrodes and HZO films are also clearly distinct. The line profiles of EELS intensities in Figure 2b further illustrate the elemental gradients and interfacial widths, confirming the precise spatial localization of the Zr‐RL. This elemental distribution analysis provides direct experimental evidence of the successful incorporation and structural confinement of the Zr‐RL within the HZO film. Figure 2c presents the cross‐sectional high‐resolution transmission electron microscopy (HR‐TEM) images of the FE capacitors with HZO/Zr‐RL/HZO stack and conventional HZO annealed at 400 °C. The insets demonstrate the (111) plane of the O‐phase with spacing of 2.94 Å in ultrathin HZO/Zr‐RL/HZO stack, whereas the conventional HZO film exhibits an amorphous nature. The bright field scanning (BF‐S) and energy dispersive X‐ray (EDS) elemental distribution maps of Hf, Zr, O and W of the FE capacitor with HZO/Zr‐RL/HZO stack and conventional HZO film are shown in Figure S2a,b (Supporting Information). Analysis of the concentration and position distribution of Hf and Zr elements also validates the presence of the Zr‐RL (Figure S2c, Supporting Information). To examine the impact of Zr‐RL on the crystallization of HZO thin films, first‐principles calculations analysis is carried out to yield atomistic insights into the phase translation in the HZO/Zr‐RL/HZO stack.^[^ ^17^, ^25^ ^]^ The simulated structure of the HZO/Zr‐RL/HZO stack used in first‐principles calculations and the energy landscape for phase transitions calculated by linear interpolation along the pathways from the tetragonal (T‐) to orthorhombic (O‐) phase and from the tetragonal to monoclinic (M‐) phase, are presented in Figure 2d,e. With the increase in tensile strain, the growth barrier for both the O‐phase and M‐phase diminishes, and the transition barrier from T‐phase to O‐phase is much lower than that from T‐phase to M‐phase. Figure 2f shows the influence of strain on the surface energy of the O‐, T‐, and M‐phases. Due to the expansion effect of Zr atoms on the crystal lattice, the ZrO_2_ crystal lattice exerts an average tensile strain of 0.76% on the HZO crystal lattice, which is beneficial for the formation of the O‐phase. Figure S3a (Supporting Information) shows the HR‐TEM image of the HZO/Zr‐RL/HZO stack film and the corresponding geometric phase analysis (GPA) derived strain maps for the region indicated by the red box. The ε_xx_ and ε_yy_ maps reveal pronounced local variations in both in‐plane and out‐of‐plane strain, indicating the presence of lattice distortion within the analyzed region, as presented in Figure S3b,c (Supporting Information). These experimentally observed strain distributions are consistent with our theoretical calculations.^[^ ^26^ ^]^ This implies that the insertion of the Zr‐RL facilitates crystallization in the HZO/Zr‐RL/HZO stacked films at low annealing temperatures. Specifically, due to the lower crystallization temperature of ZrO_2_ compared to HfO_2_, the Zr‐rich layer may crystallize preferentially, forming an initial crystalline template. Subsequently, this crystallized Zr‐rich layer could introduce additional strain to the adjacent amorphous HZO layers, compensating for the insufficient thermal strain under limited thermal budget conditions, thereby promoting crystallization into the ferroelectric phase.

### 2.3 Switching Dynamics Characterizations

Polarization switching encompasses the nucleation of the reversed domain and the domain growth. Two prevalent models, the NLS model and Kolmogorov–Avrami–Ishibashi (KAI) model are commonly used to elucidate the switching kinetics of ferroelectric materials.^[^ ^27^, ^28^ ^]^ The NLS model posits that domain wall migration occurs at a much slower rate than domain nucleation, making polarization switching predominantly constrained by the domain nucleation. This model is commonly applied to interpret phenomena in ferroelectric ceramics and polycrystalline systems.^[^ ^29^, ^30^ ^]^ To access the domain switching speed of the capacitor with the HZO/Zr‐RL/HZO stack, the frequency‐dependent *P–V* loops are initially measured under 1.0–1.5 V at various frequencies. The frequency dependence of *V*_c_ under different voltages is presented in Figure S4a–c (Supporting Information), respectively. The absolute values of ±*V*_c_ in the FE capacitor with the HZO/Zr‐RL/HZO stack exhibit an increase with the frequency, and a similar imprint effect is observed across different operation voltages (Figure S4d, Supporting Information). The linear behavior between ln *f* and 1/*V*_c_^2^ is illustrates in Figure S4e (Supporting Information), indicating that the polarization switching within the HZO/Zr‐RL/HZO stack is predominantly governed by the nucleation, which is aligned with the prediction of the NLS model.^[^ ^31^ ^]^

**Figure 3:**
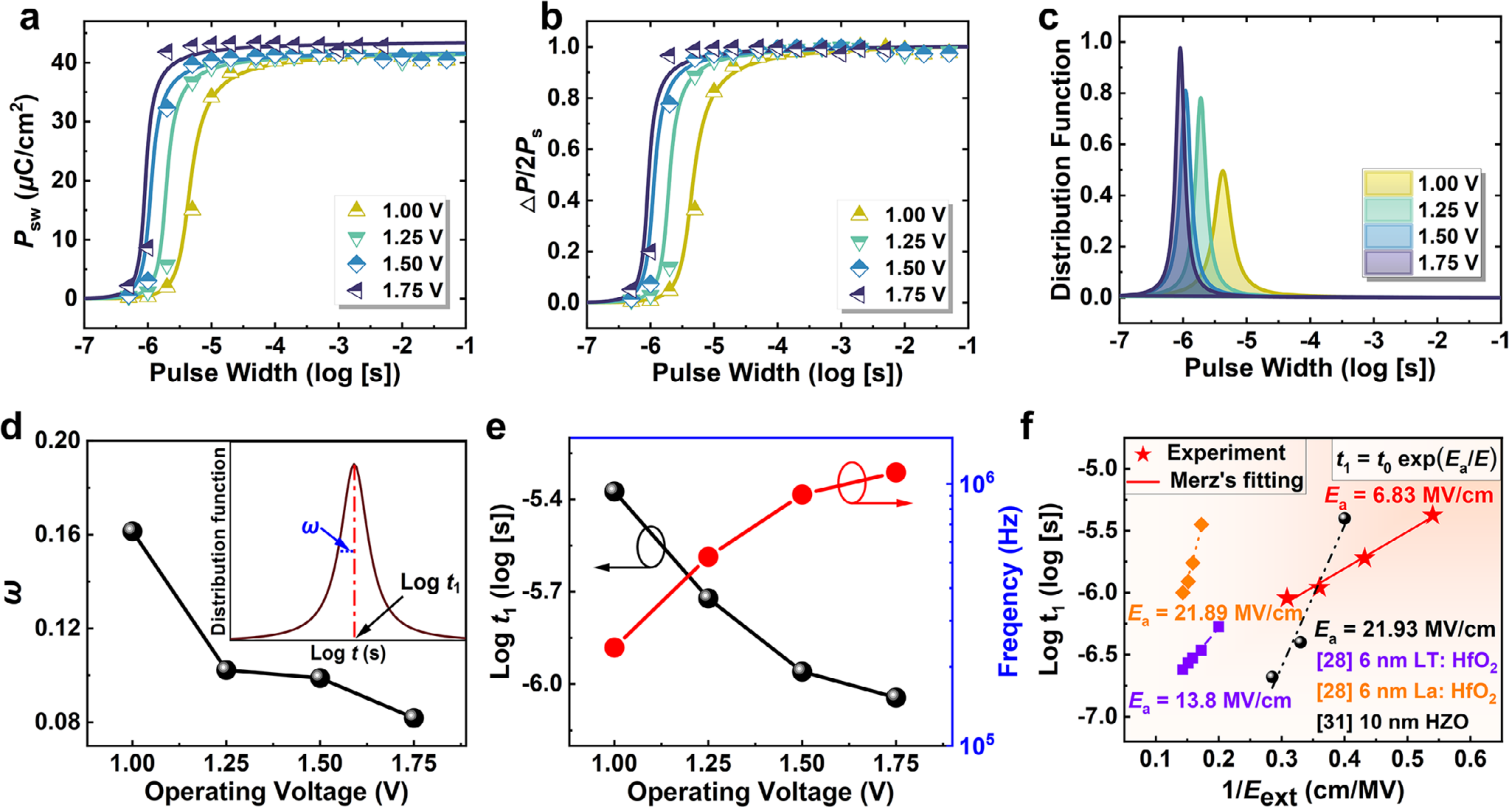
Polarization switching dynamics of the HZO/Zr‐RL/HZO stack. a) The *P*_sw_ as a function of pulse width under different *V*_op_. b) Switchable polarization measured with different amplitudes and duration time of the HZO/Zr‐RL/HZO stack. The solid lines represent the fitting results by the NLS model. c) The Lorentzian distribution functions of the HZO/Zr‐RL/HZO stack. d) The changing tendency of *ω* as a function of operating voltages. e) The extracted log *t*_1_ and corresponding switching frequency of the FE capacitor with HZO/Zr‐RL/HZO stack. f) Calculation of *E*_a_ from the field dependence of the switching time.

Furthermore, the switching dynamics of the HZO/Zr‐RL/HZO stack are evaluated via pulse switching measurements using a designed pulse train sequence, as depicted in Figure S5 (Supporting Information). **Figure** **3**a illustrates the relationship between switching polarization (*P*_sw_) and pulse width at various operating voltages. The domain switching in the HZO/Zr‐RL/HZO stack occurs at a *V*_op_ as low as 1.0 V and achieves a FE polarization of 40.44 *µ*C cm^−2^. As an increase in the operating voltage accelerates the switching speed; Specially, as the pulse amplitude reaches 1.75 V, the capacitor demonstrates rapid switching capability with a pulse width of <1 *µs*. Figure 3b displays the normalized fraction as a function of time at varying voltages, following the Lorentzian distribution functions used to fit the switching kinetics of the HZO/Zr‐RL/HZO stack (Figure 3c). The switching time for 80% polarization is recorded as 4.2, 1.9, 1.1, and 0.9 *µs* under the write voltages of 1.0, 1.25, 1.5, and 1.75 V, respectively. Besides, Figure 3d,e present the trends of the full width at half maximum, named as *ω*, and the center of the distribution (*log t*_1_), respectively. As the operating voltages increase, both *log t*_1_ and *ω* decreases, suggesting that defects, impurities, and other local potential variations in inhomogeneous systems are effectively mitigated under sufficiently strong voltages. The single pulse frequency corresponding to polarization saturation switching has been extracted, and the activation fields (*E*_a_) have been fitted by *Merz’*s empirical formula, as plotted in Figure 3f.^[^ ^32^ ^]^ Notably, an *E*_a_ of 6.8 MV cm^−1^ is demonstrated in the sub‐6 nm HZO/Zr‐RL/HZO stack, further indicating its fast switching speed and low switching field, which contributes to the corresponding low switching voltage. For comparison, the *E*_a_ reported for other ultrathin FE films and conventional 10 nm HZO is much higher than that of the Zr‐RL device, which further highlights its advantage of our Zr‐RL strategy.^[^ ^28^, ^31^ ^]^

### 2.4 Reliability Demonstration

**Figure 4:**
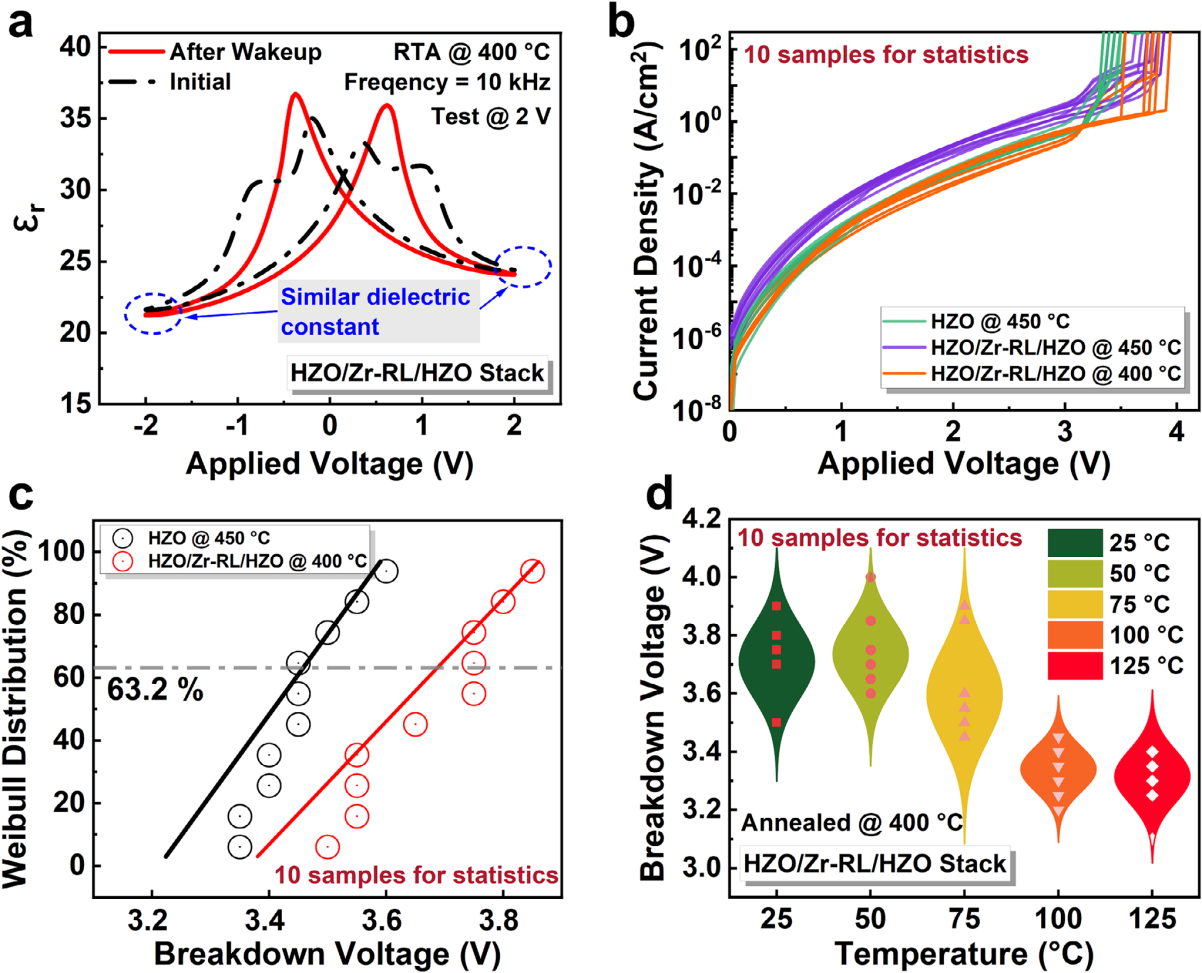
The relative dielectric constant and TZDB characteristics. a) The *ε‐V* for a capacitor with HZO/Zr‐RL/HZO stack annealed at 400 °C. b) The TZDB characteristics of capacitors with crystallized conventional HZO film annealed at 450 °C and the crystallized HZO/Zr‐RL/HZO stack annealed at 400 and 450 °C, respectively. Ten devices for each set were measured under identical conditions for statistics. c) The Weibull distribution of breakdown voltages. d) The distribution of breakdown voltages baking at different temperatures from 25 to 125 °C.

The butterfly‐shaped curves representing the relative dielectric constant‐voltage (ε_r_‐V) of the HZO/Zr‐RL/HZO stack before and after wakeup are shown in **Figures** **4**a and S6 (Supporting Information). Such distinct voltage and frequency dependence of *ε*_r_ is attributed to polarization switching in FE films under an external electric field.^[^ ^33^ ^]^ On the other hand, the convergence of peaks indicate that defect redistribution and domain depinning occurred in the film during the wakeup process.^[^ ^34^, ^35^ ^]^ The time‐zero dielectric breakdown (TZDB) of FE capacitors with conventional HZO and HZO/Zr‐RL/HZO stack film annealed at different temperatures are characterized (Figure 4b). The distribution of breakdown voltages is shown in Figure 4c. Compared to the FE capacitors with crystallized conventional HZO film, those with crystallized HZO/Zr‐RL/HZO stack annealed at 400 °C demonstrate an improved breakdown voltage up to 3.69 V at 63.2% Weibull distribution. This enhancement is primarily attributed to two factors: the reduced thermal defects resulting from lower annealing temperatures and the optimized crystalline structure, which minimizes grain boundary defects. These optimizations lead to fewer leakage paths and a delayed onset of hard breakdown.^[^ ^36^, ^37^ ^]^

The *P*–*V* loops shown in Figure S7 (Supporting Information) suggest that the capacitors retain robust ferroelectricity across the entire temperature range from 25 to 125 °C. Specifically, even at 125 °C, the 2*P*_r_ remains at 38.7 *µ*C cm^−2^ and no significant *V*_c_ shift occurs. The slight reduction in 2*P*_r_ under the low operating voltage of 1.0 V could result from the internal electric field caused by charge de‐trapping.^[^ ^38^ ^]^ Figure S8 (Supporting Information) shows the TZDB results and leakage behavior at different temperatures. The breakdown voltage distribution as a function of temperature is summarized in Figure 4d. The results reveal a moderate decrease in breakdown voltage with increasing temperature, and the capacitors maintain a breakdown voltage of 3.31 V at 125 °C. The reduction in breakdown voltage at elevated temperatures is primarily due to the increased probability of carrier injection and avalanche breakdown, leading to earlier dielectric failure. Moreover, the thermally induced defect generation and migration can locally weaken the dielectric barrier, leading to a susceptible breakdown under an applied voltage.^[^ ^39^, ^40^, ^41^ ^]^

**Figure 5:**
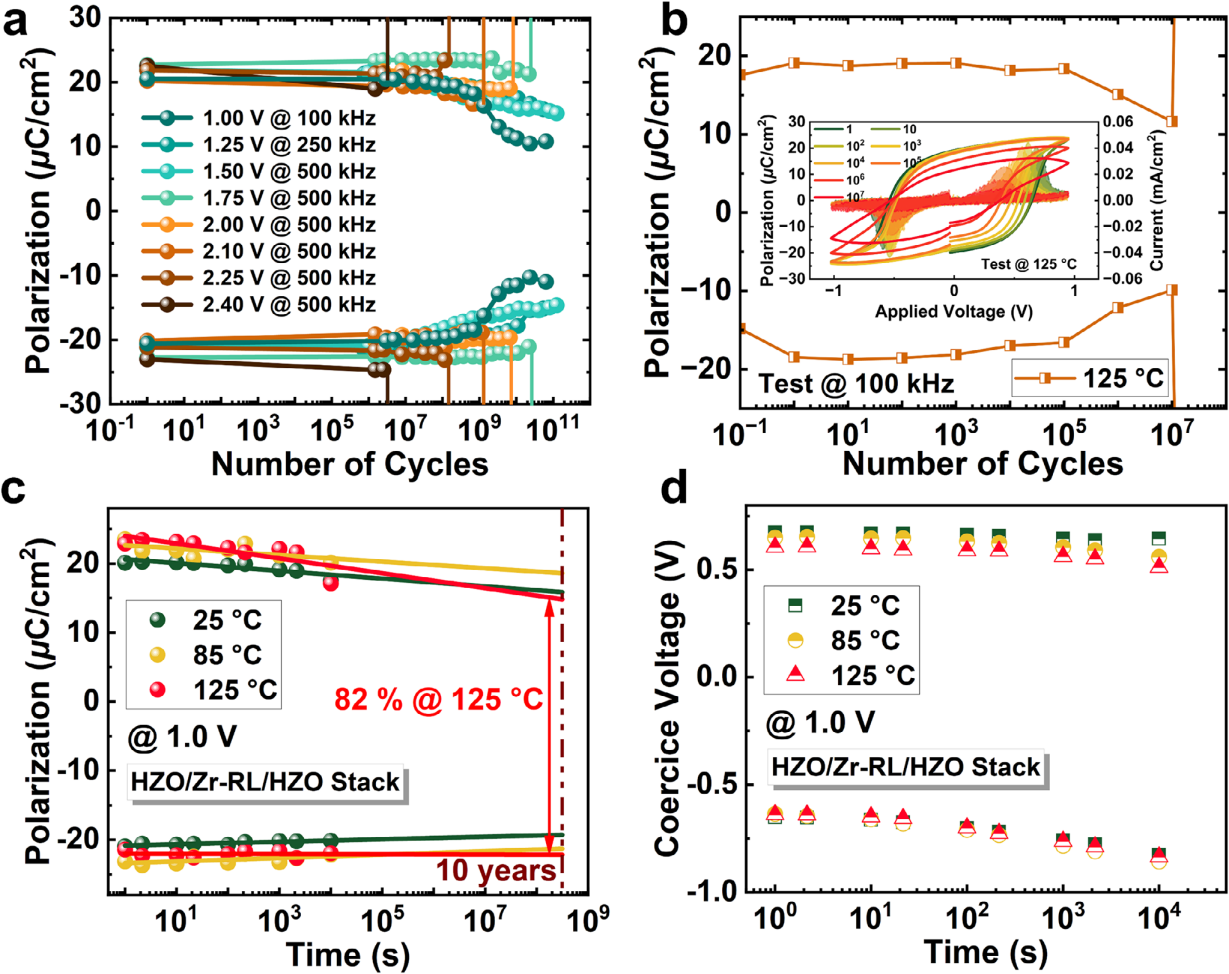
Endurance and retention characteristics of the capacitor with HZO/Zr‐RL/HZO stack. a) The cycling characteristics of the FE capacitor with *V*_op_ varying from 1.00 to 2.40 V at the saturated switching frequencies. b) Cycling characteristics of the capacitor with HZO/Zr‐RL/HZO stack under 1.0 V with a frequency of 100 kHz measured at 125 °C. The inset shows the *P*–*V* and dynamic *I‐*‐*V* curves after various cycling. c) Remanent polarization and d) coercive voltage retention for sub‐6 nm HZO/Zr‐RL/HZO stack under the *V*_op_ of 1.0 V baking at 25, 85 and 125 °C, respectively.

The reliability and retention characteristics of the capacitor with HZO/Zr‐RL/HZO stack have been demonstrated. To confirm the complete FE domain switching under applied loading pulses, different applied voltages from 1.0 to 2.4 V with saturated switching frequencies (extracted from Figure 3f) were applied to the FE capacitor. The test pulses and results are depicted in **Figures** **5**a and S9 (Supporting Information), respectively. It is observed that when the amplitude of the loading pulses exceeds 2*V*_c_, the capacitor shows hard breakdown; conversely, at lower amplitudes, a fatigue effect is observed, with no apparent correlation to the frequency of the loading pulses. Notably, the capacitor can be operated normally up to 10^11^ cycles with a considerable 2*P*_r_ when subjected to cycling voltages of at or below 1.5 V. As the applied voltages are increased beyond this threshold, the capacitor undergoes hard breakdown. Additionally, Figure S10 (Supporting Information) summarizes the relationship between the number of endurance cycles to hard breakdown and the operating voltages under a saturated frequency of 100 kHz. The number of endurance cycles is found to decrease by one order of magnitude for every increase of 0.22 V, and the breakdown‐limited endurance of the FE capacitor with HZO/Zr‐RL/HZO stack operating at 1.0 V is estimated to be ≈10^11^ cycles. The cycling endurance of the HZO/Zr‐RL/HZO stack capacitor under 1.0 V with a frequency of 100 kHz was evaluated at 125 °C, as displayed in Figure 5b. The capacitor exhibits robust ferroelectric switching behavior and maintains a considerable 2*P*_r_ over 20 *µ*C cm^−2^ even after 10^7^ cycles at 125 °C. The inset *P*–*V* and dynamic *I*–*V* curves, along with the leakage current shown in Figure S11 (Supporting Information) further confirm the preservation of ferroelectric characteristics and the absence of hard breakdown up to 10^7^ cycles at 125 °C.

Figure 5c,d shows the time evolution of *P*_r_ and *V*_c_ for HZO/Zr‐RL/HZO FE capacitors measured at different temperatures of 25, 85, and 125 °C under the *V*_op_ of 1.0 V. Figure S7 (Supporting Information) illustrates the waveform settings used for the retention measurement. After applying a write pulse and a predetermined delay period, the PUND method was employed to determine the remanent polarization of the capacitor. Figure S13 (Supporting Information) presents the *P*–*V* loops measured at various time intervals after the write operation during retention tests. The excellent retention performance is demonstrated at all tested temperatures, maintaining considerable polarization after extrapolation to 10 years. In particular, it predicted the ≈82% retention of initial polarization after 10 years at 125 °C. As shown in Figure 5d and Figure S7 (Supporting Information), the negative *V*_c_ has a notable shift at all temperatures, whereas the positive *V*_c_ remains relatively stable at 25 °C, while exhibits a decrease at 85 and 125 °C. This asymmetric *V*_c_ shift indicates the presence of imprint effects, which result from the asymmetric internal electric field induced by charge trapping or defect accumulation at the electrode‐ferroelectric interface during prolonged retention measurements.^[^ ^42^, ^43^, ^44^ ^]^ In Table S1 (Supporting Information), a comparison of the performance achieved by the Zr‐RL strategy with other previously proposed optimization approaches is provided.^[^ ^7^, ^10^, ^23^, ^24^, ^45^ ^]^ It is observed that the ferroelectric capacitors with ultrathin FE film fabricated using the Zr‐RL strategy exhibit the highest reliability, extremely low operating voltages, and outstanding ferroelectric properties. This demonstrates that the Zr‐RL structure effectively mitigates the performance degradation and increased thermal budget issues encountered during the thickness scaling of HZO films, accordingly offering a promising solution for BEOL‐compatible FeRAM applications in advanced process nodes.

## 3 Conclusion

In summary, the zirconium‐rich layer strategy is systematically investigated in terms of ferroelectric properties, microstructure, switching kinetics, and reliability. Compared to the FE capacitor with conventional HZO film, the FE capacitor with sub‐6 nm HZO/Zr‐RL/HZO stack exhibits a significantly low annealing temperature of 400 °C, an ultra‐low *V*_op_ of 1.0 V, and an excellent ferroelectricity with a saturated 2*P*_r_ of 43.4 *µ*C cm^−2^. First‐principles calculations reveal at the atomistic level that the Zr‐RL could introduce the additional tensile strain and reduce the growth barriers of the ferroelectric phase. Furthermore, the FE capacitor with HZO/Zr‐RL/HZO stack demonstrates superior reliability, evidenced by a high breakdown voltage of 3.69 V and endurance characteristics exceeding 10^11^ cycles. In terms of the thickness scaling and process temperature, the zirconium‐rich layer strategy is demonstrated to be an innovative approach to optimizing both the ferroelectricity and reliability toward BEOL‐FeRAM applications.

## 4 Experimental Section

### Device Fabrication

A 30 nm tungsten (W) was deposited onto the Si/SiO_2_ substrate by a magnetron sputtering system (PVD 75, Kurt. J. Lesker) at room temperature. Then, a 2.2 nm HZO/1 nm ZrO_2_/2.2 nm HZO stack was grown by PEALD (TFS 200, Beneq) at 250 °C utilizing TEMAH, TEMAZ, and oxygen plasma as the Hf, Zr, and O precursors, respectively. In addition, a capacitor with ≈5.4 nm conventional HZO film was also prepared for comparison. Subsequently, a 30 nm W was sputtered by magnetron sputtering system, photo‐lithography, and wet etching in sequence to form top electrode with an area of 80 × 80 *µm* ^2^. Finally, the FE capacitors are annealed at 400 and 450 °C in nitrogen atmosphere (N_2_) for 60 s using rapid thermal annealing.

### Electron Microscopy Characterizations

The cross‐sectional HR‐TEM lamella was prepared by using a focused ion beam (FIB, Helios G4, Thermofisher) at 30 kV, and followed by a final ion cleaning process performed at 5 kV to reduce the surface damage layer of the TEM lamella. The final TEM lamella thickness was ≈50 nm. HR‐TEM and STEM analyses were conducted using a Talos F200x (ThermoFisher Scientific) operating at an accelerating voltage of 200 kV. For high‐resolution STEM (HR‐STEM) imaging, a convergent semi‐angle of ≈100 mrad and a probe diameter of ≈0.16 nm are employed, enabling atomic‐scale resolution.

### Electrical Measurements

Electrical measurements were implemented via a metal‐ferroelectric‐metal (MFM) capacitor, using a Radiant Workstation ferroelectric tester in conjunction with an Agilent B1500 semiconductor parameter analyzer. Throughout all electrical measurements, a bias voltage was applied to the bottom electrode, and the top electrode is grounded. Various measurement techniques were employed to investigate the macroscopic electrical properties, including PUND tests, time‐zero dielectric breakdown analysis, capacitance‐voltage (C‐V) characteristics, switching dynamics, endurance, and as well as retention characteristics.

### First‐Principles Calculations

All first‐principles calculations were performed by density‐functional theory (DFT) implemented in the QuantumATK simulation platform. The exchange‐correlation functional of Perdew–Burke–Ernzerhof (PBE) at generalized gradient approximation (GGA) and the SG15 Optimized Norm‐Conserving Vanderbilt pseudopotential (ONCVPSP) were used for all calculational analyses. The density mesh cutoff was 140 Har and the force tolerance was set as 0.01 eV Å^−1^ for structural optimization. For the unit‐cell calculation, the Brillouin zone was sampled with 5 × 5 × 5 k‐points grid. The energetically favorable supercell model was constructed with Zr and Hf layers emerging alternately along the *C*‐axis, consistent with the experimental growth realized by ALD. To take surface energy into account to the investigation of the strain effect, the slab models were built with a 6 unit‐cell (UC) HZO in the C direction and 3UC vacuum at both ends. The sampling k‐points were set to be 5 × 5 × 1 for the slab‐model relaxation. Atoms at the surfaces are relaxed and those in the central regions are fixed to prevent the drift of the slab and calculate the relaxation energy. The biaxial in‐plane tensile strain from 1% to 3% was applied on the crystal plane of T‐(010)/O‐(001)/M‐(100), while the polarization direction was optimized to align with ferroelectric phase. It's noted that excess biaxial strain transforms the T‐phase (1%) and O‐phase (2%) into a paraelectric phase with a space group of Pbcn. The energy landscape for phase transformation along the pathways of T → O and T → M was determined using the variable‐cell nudge elastic band (vc‐NEB) method. Moreover, the minimum‐energy images of intermediate structures were generated by the linear interpolation method in the unit cell. For the convergence of vc‐NEB, the minimum energy paths were calculated with a force tolerance of 0.05 eV Å^−1^. And the spring constants between neighboring images were set in the range of 5.0 eV Å^−3^.

## Conflict of Interest

The authors declare no conflict of interest.

## Supporting information

Supporting Information

## Data Availability

The data that support the findings of this study are available in the supplementary material of this article.
