# Supplementary material for: Zirconium‐Rich Strategy in Ultrathin Hf0.5Zr0.5O2 toward Back‐End‐of‐Line‐Compatible Ferroelectric Random Access Memory
Source: Adv Sci (Weinh). 2025 Aug 14;12(42):e09384. doi: 10.1002/advs.202509384 (PMC12622460; doi:10.1002/advs.202509384)
Supplement: Supplementary file 1 — Supporting Information [file ADVS-12-e09384-s001.pdf]

Supporting Information

Zirconium-Rich Strategy in Ultrathin $Hf_{0.5}Zr_{0.5}O_2$ toward Back-End-of-Line-Compatible Ferroelectric Random Access Memory

Yinchi Liu[1,2], Jiajia Tao[3], Xiaoyu Dou[4], Kangli Xu[1,2], Yuchun Li[2], Handong Zhu[1,2], Hongliang Lu[2], Yanxi Li[2], Chi Liu[5], Jiezhi Chen[4], Lin Chen[1,2], Shijin Ding[2], Jixuan Wu[4*], and Wenjun Liu[1,2,6*]*

[1] College of Integrated Circuits and Micro-Nano Electronics, Fudan University, Shanghai 200433, China

[2] School of Microelectronics, Fudan University, Shanghai 200433, China

[3] Zhangjiang Laboratory, Shanghai 201210, China

[4] School of Information Science and Engineering, Shandong University, Jinan 250100, China

[5] Shenyang National Laboratory for Materials Science, Institute of Metal Research, Chinese Academy of Sciences, Shenyang 110000, China

[6] Shaoxin Laboratory, Shaoxing 312000, China

*Corresponding authors, E-mails: jixuanwu@sdu.edu.cn, linchen@fudan.edu.cn, wjliu@fudan.edu.cn

Fabrication and device structures

Figure S1a and S1b display the process flows of FE capacitors with conventional HZO and 2.2 nm HZO/1 nm ZrO_2/2.2 nm HZO stack and atomic layer deposition (ALD) pulse cycles, respectively. During the deposition process of the stacked films, the introduction of the Zr-rich layer is achieved by controlling the pulses of HfO_2 and ZrO_2. Wherein, Tetrakis(dimethylamido)hafnium (@55°C), Tetrakis(dimethylamido)zirconium (@65°C), and oxygen plasma were utilized as the Hf, Zr, and O precursors, respectively.

Figure S2a-S2c show the bright field scanning (BF-S) and energy dispersive X-ray (EDS) elemental distribution maps of Hf, Zr, O and W of the FE capacitor with HZO/Zr-RL/HZO stack and the conventional HZO film. An obvious Zr-RL was observed in the HZO/Zr-RL/HZO stack, which arises from the differences in the positioning and composition of Hf and Zr elements. The results from EDS line scan further substantiate this observation. Additionally, the interdiffusion between Zr and Hf elements was seen, which is attributed to the heat treatment processes, including both the deposition and annealing stages. Therefore, even though the design during the deposition process aims to create a pure ZrO_2 layer in the middle of the ferroelectric (FE) film (meaning no Hf elements), this objective proves to be extremely

challenging due to the influence of post-metallization annealing. This is also a primary reason for naming the layer as Zr-RL.

Figure S3a displays a HR-TEM image, where the red box marks the area subjected to further strain analysis. Geometric phase analysis (GPA) was carried out using the DigitalMicrograph software. Specifically, two non-collinear g-vectors corresponding to in-plane and out-of-plane lattice directions were selected from the FFT pattern of the marked region. For the strain mapping, two non-collinear g-vectors were selected from the FFT pattern of the marked region: the x-plane (in-plane) direction corresponds to the orthorhombic (O-) phase (200) reflection with a lattice spacing of 2.45 Å, and the y-plane (out-of-plane) direction corresponds to the O-phase (02-2) reflection with a lattice spacing of 1.76 Å.[1] Circular masks were applied to isolate these diffraction spots, and the resulting phase images were used to calculate the local strain component ε_{xx} and ε_{yy}, as shown in Figures S3b and S3c, respectively. These color-coded strain maps reveal pronounced spatial variations in both in-plane and out-of-plane strain, confirming the existence of local lattice distortion in the vicinity of the Zr-rich layer.[2]

Frequency-dependent and switching dynamics of the capacitor with HZO/Zr-RL/HZO stack

The frequency-dependent of P-V hysteresis loops under different operating voltages (V_{op}) of the capacitor with HZO/Zr-RL/HZO stack is characterized as shown in Figure S4a-S4c. As the testing frequency increases, the coercive voltages (V_c) initially remain stable and then gradually increase with further rise in frequency. This is mainly due to the difficulty of ferroelectric domain switching at high frequencies. The frequency-dependent V_c is illustrated in Figure S4d. Notably, a similar frequency-dependent behavior is observed at an V_{op} of 1.0 V, indicating that FE domains can maintain stable switching even at 1.0 V. The relationship between V_c and frequency is deducted from Equation (1),

$$\ln f = \ln f_0 - \frac{\alpha}{k_B T} * \frac{1}{V_c^2} \qquad (1)$$

Here, the f_0 is the limiting switching frequency at a high applied voltage, where nucleation is no longer the rate-controlling step. The parameter α is related to domain wall energy and the ratio of a switching polarization by the nucleus. The k_B and T are the Boltzmann constant and the temperature, respectively. Fitting the experimental data to this model reveals a distinct linear relationship between ln f and $1/V_c^2$, as shown in Figure S4e, indicating that the FE domain switching in the HZO/Zr-RL/HZO stack film conforms to the nucleation-limited switching (NLS) model.

Figure S5 depicts the waveform configuration for measuring the switching dynamics of the FE capacitor with HZO/Zr-RL/HZO stack. The waveform could be divided into three parts. Firstly, a preset pulse is applied on the FE capacitor for ensuring the full polarization and refreshing the capacitor. Secondly, a write pulse is applied to investigate the relationship among polarization switching, voltage, and pulse duration. Thirdly, the switching polarization (P_{sw}) is determined through the application of two consecutive read pulses. The charge measured by the first read pulse includes both domain switching and dielectric response, while the second pulse measures exclusively the dielectric response. By subtracting the values obtained from the second pulse from those of the first, the FE switching current can be isolated, allowing for the P_{sw} ultimately calculation.

Dielectric constant and reliability measurements.

Figure S6a and S6b show the relative dielectric constant (ε_r) before and after wakeup as a function of applied voltage, respectively. Both the butterfly-shaped curves of the ε_r-V, obtained before and after wakeup under different frequencies are obtained, indicating the ferroelectricity of the FE capacitor with the HZO/Zr-RL/HZO stack.

Figure S7 presents the temperature-dependent *P–V* hysteresis loops for the capacitor with the HZO/Zr-RL/HZO stack. Measurements were conducted at the V_{op} of 1.0 V, with the capacitor subjected to temperatures ranging from 25 to 125 °C. All loops were recorded after electrical stabilization to minimize wake-up effects and ensure consistent switching behavior.

Figure S8 shows the time-zero dielectric breakdown (TZDB) characteristics of the capacitor with HZO/Zr-RL/HZO stack across a range of temperatures (25 °C to 125 °C). For each temperature, measurements were carried out on ten statistically independent capacitors to ensure reproducibility. During measurement, the applied voltage was incrementally increased, and the current density was monitored to capture the onset of dielectric breakdown. The data reveal that increasing the ambient temperature leads to higher leakage currents and a gradual decrease in breakdown voltage. Nevertheless, the capacitors consistently maintain good dielectric integrity throughout the tested temperature window, further supporting the structural robustness of the HZO/Zr-RL/HZO stack design.

Figure S9 presents the waveform for endurance measurement, which can be divided into three distinct phases, denoting as wakeup, cycling, and positive-up-negative-down (PUND). To more accurately assess the relationship between FE polarization and pulse cycles, a wakeup process with 10^3 pulses is performed prior to the cycling phase, using a pulse amplitude of 1.5 V and a frequency of 1 kHz. Subsequently, based on the switching times of the HZO/Zr-

RL/HZO stack at different voltages extracted from the switching dynamics, we investigate the relationship between remnant polarization (P_r) and cycling times. And, the P_r after cycling is obtained using the PUND method.

Figure S10 summarizes the endurance performance of the HZO/Zr-RL/HZO stack capacitors tested at 100 kHz under various V_{op} ranging from 1.70 to 2.30 V. The polarization retention was continuously monitored throughout cycling, and the abrupt loss of switchable polarization was used to define device failure. In Figure S10b, the measured endurance data were fitted using a linear regression on a semi-logarithmic scale, with both 95 % confidence and prediction bands indicated. This approach allows for a quantitative assessment of voltage-accelerated degradation, confirming the robust cycling stability of the stack over a wide range of conditions at room temperature.

Figure S11 presents the leakage current density evolution of the HZO/Zr-RL/HZO capacitor measured at 125 °C as a function of cycling number. The leakage was recorded by sweeping the applied voltage in both directions at specified cycle intervals, using the same device for all measurements. The results indicate only a minor increase in leakage current even after extensive cycling at elevated temperature, further supporting the excellent endurance and electrical reliability of the HZO/Zr-RL/HZO stack under high temperature conditions.

Retention characteristics.

Figure S12 illustrates the pulse waveform scheme used in the retention measurement. Initially, a "Write" pulse (1.0 V, width of 0.5 ms) is applied to preset the ferroelectric state of the capacitor without measurement during this step. Subsequently, after a variable delay (1~10000 s in the measurement), a positive read pulse labeled "x" is applied. Under this positive pulse, no polarization switching occurs, thereby capturing the non-effective remanent polarization state. Following an additional delay of 1 s to allow the decay of non-effective remanent polarization, a positive pulse labeled "U" is applied. This pulse does not induce polarization switching and thus captures the non-effective remanent polarization signal. Thereafter, a negative pulse ("N") is applied, resulting in polarization reversal from the positive back to the negative state, enabling the recording of the polarization switching characteristics in the opposite direction. After another 1 s delay, a negative pulse ("D") without polarization switching is applied to measure the corresponding non-effective remanent polarization in the negative direction. Finally, a positive pulse ("P") is applied again to induce polarization switching from negative to positive, completing the polarization cycle. By subtracting the polarization responses of these pulses (P minus U, and N minus D), the genuine remanent

polarization hysteresis loop can be accurately determined. This testing approach ensures precise extraction of the effective polarization state by systematically removing non-switching polarization contributions, thereby providing reliable retention characteristics of the ferroelectric capacitors. Figure S13 shows the P-V loops of the FE capacitors with HZO/Zr-RL/HZO stack under the operation voltage of 1.0 V baking at different temperatures from 25 to 125 °C.

Finally, we compare the performance achieved by the Zr-RL strategy with other previously proposed optimization approaches. [1,2,3,4,5] It is observed that the ferroelectric capacitors with ultrathin FE film fabricated using the Zr-RL strategy exhibit the highest reliability, extremely low operating voltage, and outstanding ferroelectric properties. This demonstrates that the Zr-RL structure effectively mitigates the performance degradation and increased thermal budget issues encountered during the thickness scaling of HZO films, offering a promising solution for BEOL-compatible FeRAM applications in advanced process nodes.

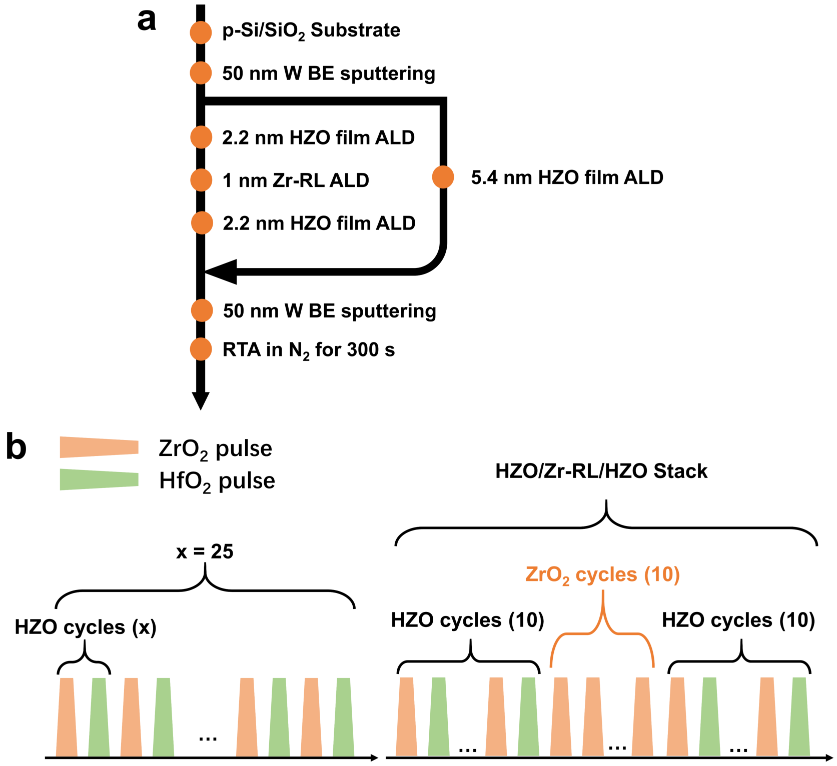

Figure S1. The fabrication of the FE capacitors. a) Key process flow of the FE capacitors with conventional HZO and HZO/Zr-RL/HZO stack. b**)** ALD cycles of ZrO_2 and HfO_2 pulse during the growth of conventional HZO and HZO/Zr-RL/HZO stack.

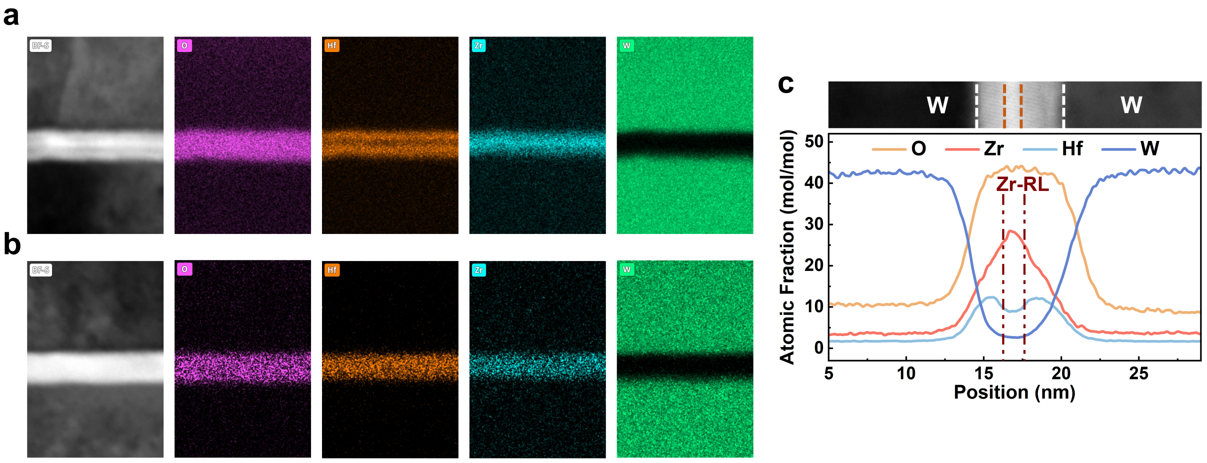

Figure S2. The device structures and elements distribution. The BF-S and EDS results for the distribution of O, Hf, Zr and W atoms in the capacitor with a) HZO/Zr-RL/HZO stack and b) conventional HZO. c) EDS line trace for FE capacitor with HZO/Zr-RL/HZO stack.

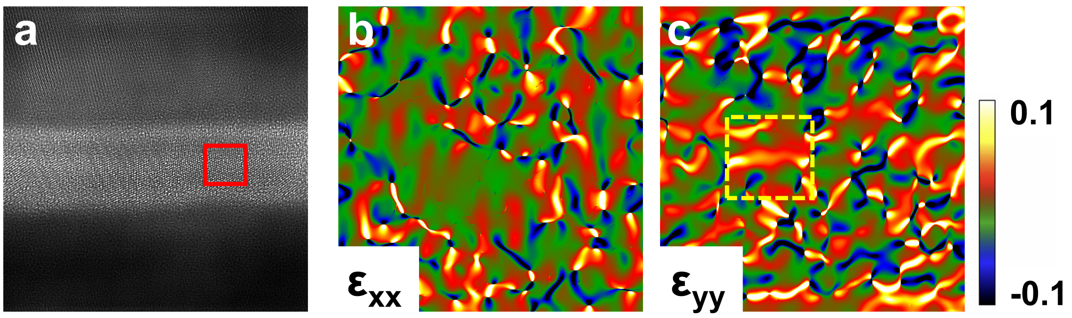

Figure S3. a) HR-TEM image of the HZO/Zr-RL/HZO stack film. The red box indicates the region selected for GPA analysis. b)-c) Strain maps (ε_{xx} and ε_{yy}) derived from GPA of the corresponding area, revealing local strain distributions within the stack.

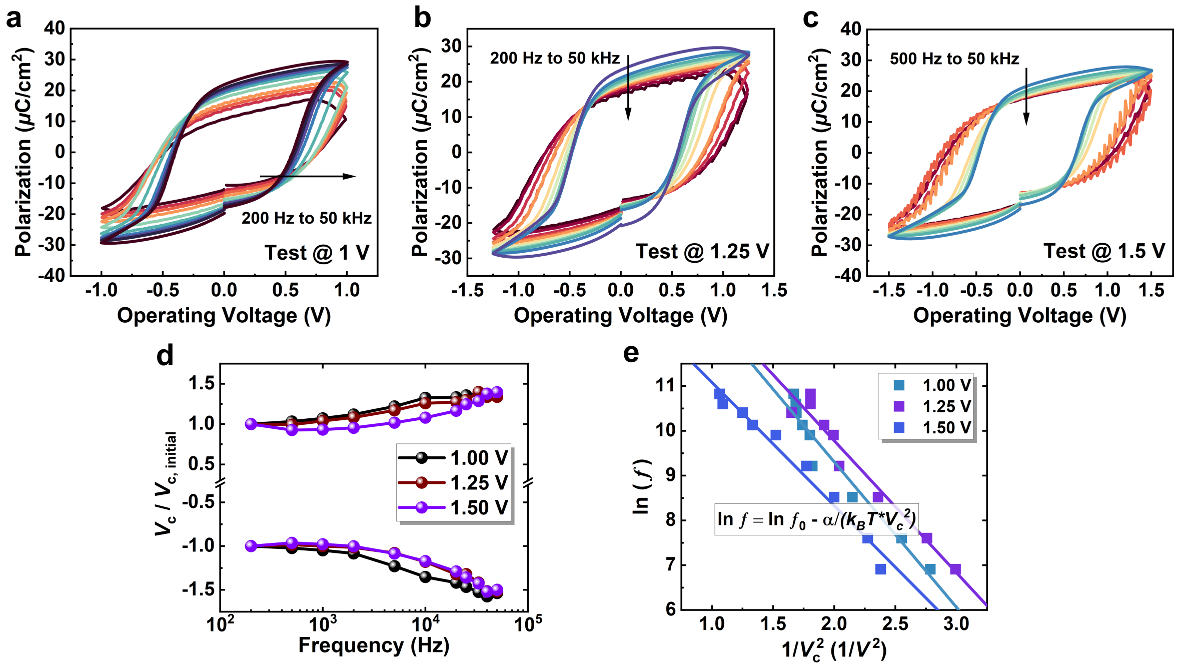

Figure S4. Frequency-dependent *P*-*V* loops of the capacitor with the HZO/Zr-RL/HZO stack. The *P*-*V* curves of FE capacitor with the HZO/Zr-RL/HZO stack under a**)** 1.0, b) 1.25 and c) 1.5 V under different frequencies. d) The frequency-dependent of V_c under different operating voltages. e) lnf versus $1/V_c^2$ curves of the FE capacitor with HZO/Zr-RL/HZO stack.

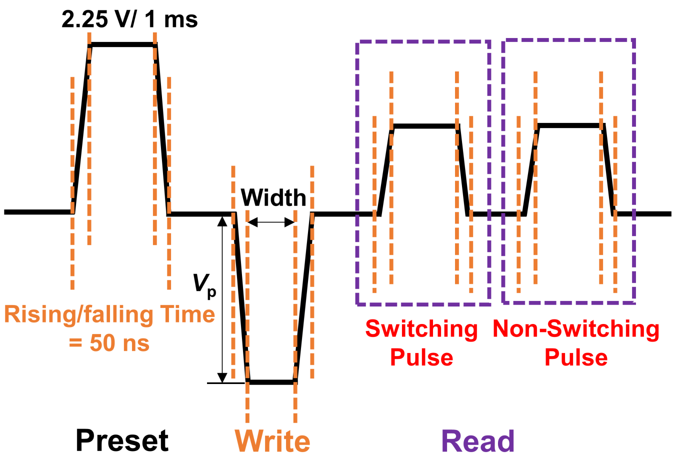

Figure S5. Pulse sequence used for switching dynamic measurement.

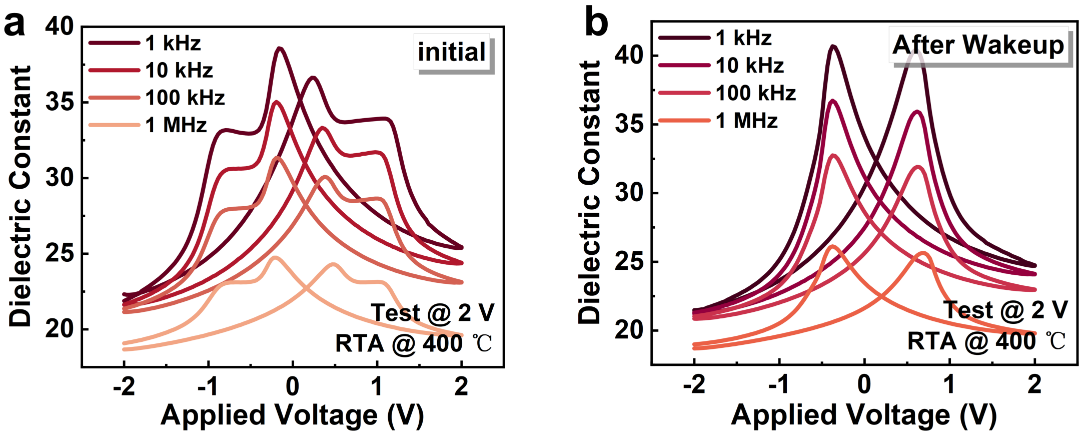

Figure S6. The relative dielectric constant of the FE capacitor with the HZO/Zr-RL/HZO stack. The relative dielectric constant a) before and b) after wakeup under different frequencies from 1 to 1000 kHz. Both the dielectric constants before and wakeup are measured under the voltage of 2 V.

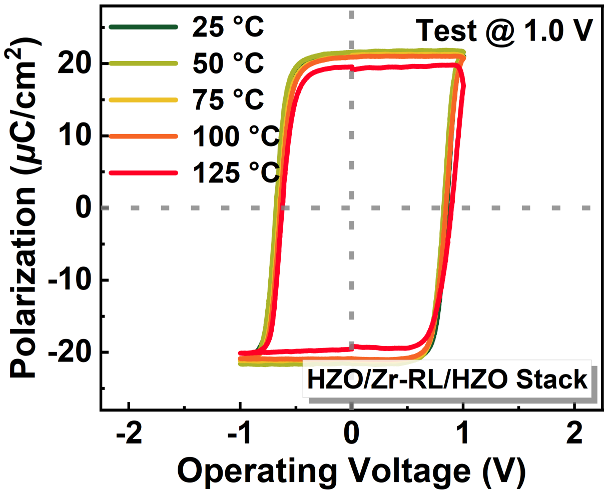

Figure S7. The *P*-*V* loops of the capacitor with HZO/Zr-RL/HZO stack measured at different temperatures from 25 to 125 °C.

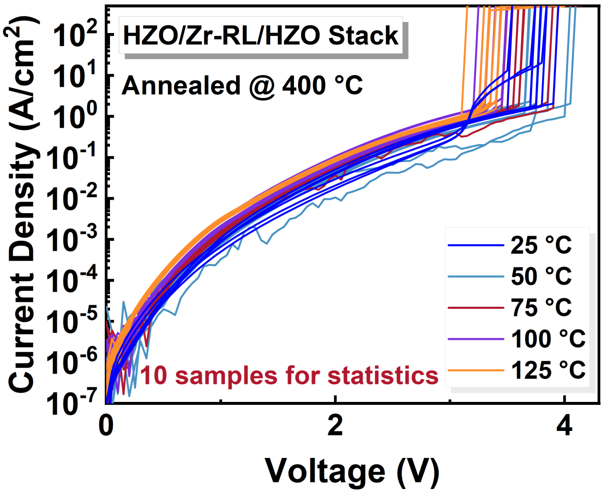

Figure S8. The TZDB characteristics of the capacitor with HZO/Zr-RL/HZO stack measured at different temperatures from 25 to 125 °C.

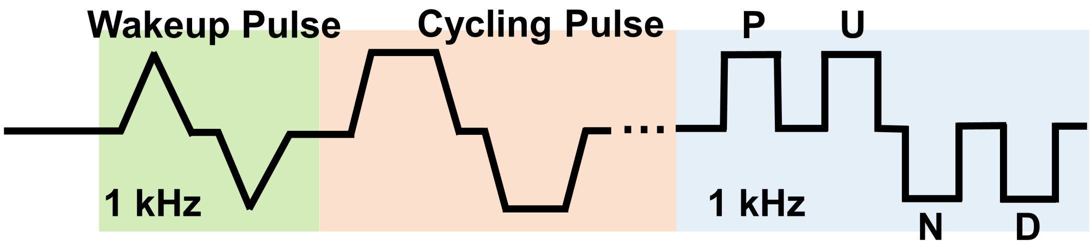

Figure S9. The waveform setting for endurance measurement.

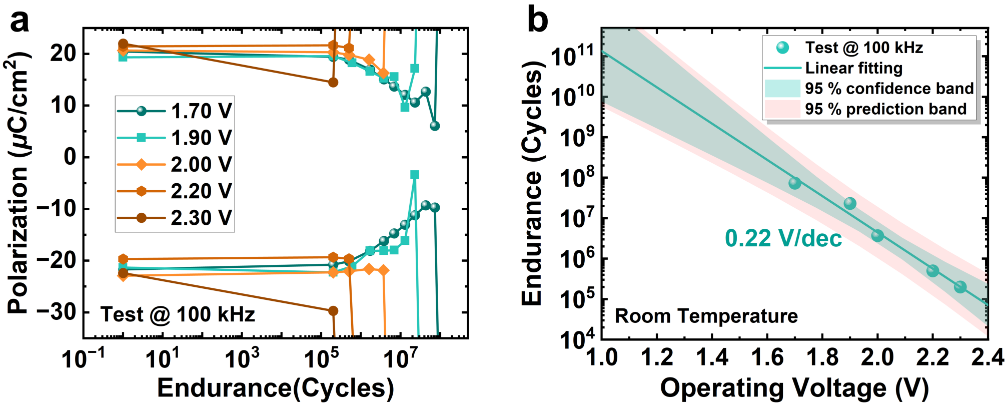

Figure S10. The endurance cycles of capacitor with HZO/Zr-RL/HZO stack measured at different voltages from 1.7 to 2.3 V with a frequency of 100 kHz.

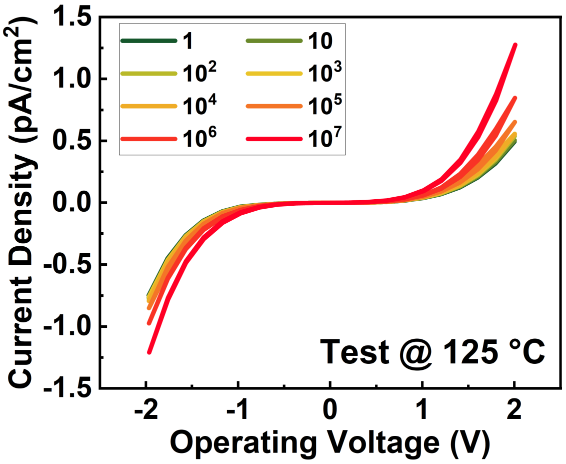

Figure S11. Leakage current density of the HZO/Zr-RL/HZO stack capacitor measured at 125 °C after different cycle numbers.

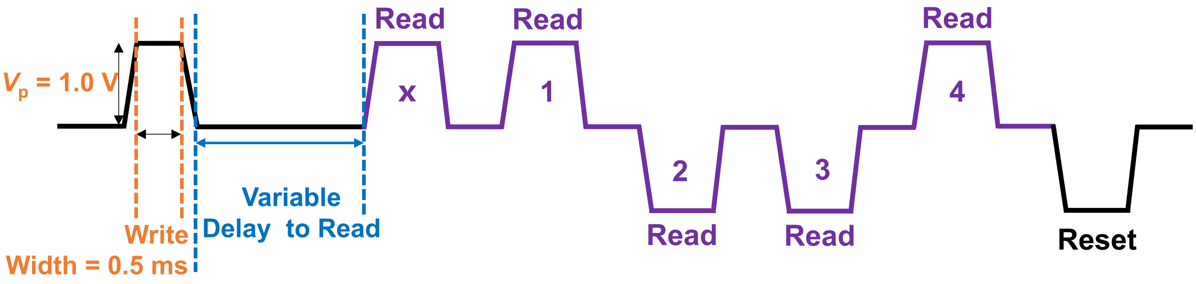

Figure S12. The waveform setting for retention measurement.

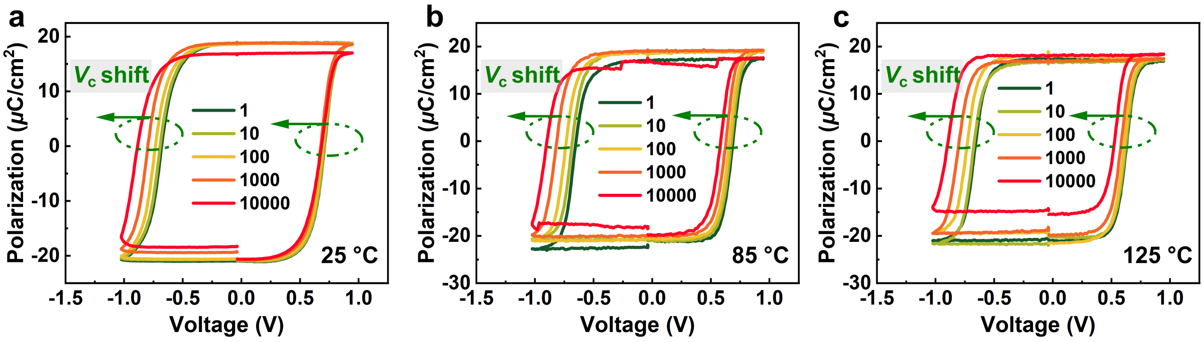

Figure S13. *P-V* curves of the capacitors with HZO/Zr-RL/HZO stack under the V_{op} of 1.0 V baking at (a) 25, (b) 85 and (c) 125 °C measured at various time intervals after the write operation during retention measurements.

Table 1 Benchmark of FE capacitors with ultrathin HZO film

Total Thickness	Strategy	V_{op}	$2P_r$	Max. Endurance cycles	Annealing Temp.	Ref.
**~5.4 nm**	**Zirconium-rich layer**	**1.0 V**	**43.4 μC/cm^2**	**projected to 2e10^{11}**	**400 °C**	**This work**

~8.0 nm	Pre-crystallization engineering	2.0 V	27 μC/cm^2	projected to 10^{12}	400 °C	[1]
~5.5 nm	Interfacial layer	1.5 V	15 μC/cm^2	> 10^{10}	350 °C	[3]
5 nm	Thermal/Plasma enhanced	2.0 V	38.5 μC/cm^2	> 10^{10}	400 °C	[4]
4 nm	con. HZO	1.6 V	39 μC/cm^2	projected to 10^{14}	500 °C	[5]
6 nm	Superlattice	2.0 V	30 μC/cm^2	~ 10^{10}	600 °C	[6]

Reference

[1] P. Jiang, H. Jiang, Y. Yang, L. Tai, W. Wei, T. Gong, Y. Wang, P. Xu, S. Lv, B. Wang, J. Gao, J. Li, J. Luo, J. Yang, Q. Luo, M. Liu, In *2021 IEEE International Electron Devices Meeting (IEDM)*, **2023**.

[2] K. Cao, Q. Zhao, J. Liao, F. Yan, K. Bao, S. Jia, J. Zhang, J. Luo, M. Liao, Y. Zhou, *Microstructures* **2025**, *5*.

[3] E. Yu, X. Lyu, M. Si, P. D. Ye, K. Roy, *IEEE Transactions on Electron Devices* **2023**, 70, 2962.

[4] C.-H. Chien, Y-C. Huang, S-J. Chang, S-Y. Wang, Y-H. Lin, C-H. Chien, *IEEE Transactions on Nanotechnology* **2024** *23*, 471.

[5] K. Tahara, K. Toprasertpong, Y. Hikosaka, K. Nakamura, H. Saito, M. Takenaka, S. Takagi, *2021 IEEE Symposium on VLSI Technology and Circuits (VLSI Technology and Circuits)* **2021**, T7-3.

[6] Y.-K. Liang, Z. Liu, Z. Cai, X. Han, H.-Y. Huang, Y.-M. Lin, E. Yi Chang, C.-H. Lin, M. Takenaka, K. Toprasertpong, S. Takagi, *IEEE Electron Device Letters* **2024**, 45, 1468.
